# Strengthen and Respect Each Thread

**DOI:** 10.3390/ijerph192114117

**Published:** 2022-10-29

**Authors:** Virginia Araceli Feliz, Sue D. Hobbs, Rose Borunda

**Affiliations:** College of Education, California State University, Sacramento, CA 95819, USA

**Keywords:** diversity, community, inclusion, mental health, representation

## Abstract

Through a culturally grounded epistemology, this article provides mental health practitioners and researchers an overview of how generational trauma can impact the well-being of Black, Indigenous, Latinx, and other historically marginalized communities. Historically, deficit-based lenses frame the experiences of Black, Indigenous, and other People of Color (BIPOC). Discussion of white supremacy as a factor that creates divisiveness, discontinuity, and othering is necessary to understand mental healthcare for marginalized communities. Research has shown that behaviors, identities, and expressions that are respected in indigenous cultures and communities are most often misrepresented, ignored, erased, and ultimately misidentified as requiring rehabilitation. In fact, researchers assert that the organizational culture of the mental health industry limits access for minoritized communities due to lack of practitioner relational capacity, and inclusive practices. This article illustrates examples of white supremist practices through Native American storytelling to trace generational trauma from its origins, when Eurocentric perspectives were imposed upon America’s original inhabitants, to trauma caused by placement of BIPOC children in the foster care and adoption system. While fully aware of the complexities of mental health care, the authors argue that diverse cultural representations of identity, knowledge, and collectivism should inform mental health practice, and research.

## 1. Introduction

*Humankind has not woven the web of life. We are but one thread within it. Whatever we do to the web, we do to ourselves. All things are bound together. All things connect.*

—Chief Seattle, Duwamish (1780–1866) [1]

A frayed community’s destiny is like a compromised piece of fabric. When a thread unravels from the whole, the integrity of the entire piece is threatened. Over time, the threads surrounding the compromised thread strain with their attempt to compensate for the weakened state of the whole. With considerable pressure on the whole, the piece may disassemble because it lacks the collective strength to sustain external pressure. Ultimately, the collapse of the whole is attributable to one unstable thread. In this analogy of a thread and fabric (the whole), the fabric represents the communities of Black, Indigenous, and People of Color (BIPOC) while the unraveling thread represents white supremacy as a factor that contributes to the disintegration of society. 

With this understanding of how the weakened state of one can destabilize the whole, we cast light on the strength and health of our society and how the loose thread which we refer to as white supremacy has led to compromising the whole. A brief examination of our community fabric reveals that the fray, left loose, compromises the whole. If there are members affected by this loose thread whose needs are unattended and they, themselves, are unvalued, the collective community pays a price. More so, the reasons for which the threads of our community have been cast outside the body speaks more about the community than the threads themselves. A body that knowingly compromises itself is essentially pathological.

Educators, mental/physical health practitioners, policy makers, and a host of others who attend to quality of life have considerable concern about the health and well-being of its community members. Sachs indicates that despite a long term rise in U.S. income per person that there are, nonetheless, evident “trends adverse to subjective well-being: worsening health conditions for much of the population; declining social trust; and declining confidence in government” [2] (p. 125). While the cause of such discontent has been studied, we are left to wonder why a nation with such vast material wealth would see sweeping prevalence of addictions that include opioids, internet, eating, and a host of other devolving maladies. Notably, these epidemics are “accompanied by rising suicide rates and overdoses related to substance abuse, rising obesity related to eating addictions, and rising adolescent depression apparently related to Internet and related addictions” [2] (p. 128). The level of discontent is not only palpable, it is self-imploding.

What has come to bear upon the fabric of U.S. society and the communities within is an unraveling of human civility and interrelatedness whose origins can be traced to the devaluation of those ‘othered’ during the European Diaspora. The subsequent violent imposition of one cultural view has been and continues to be propelled by forces of the Doctrine of Discovery [3], Manifest Destiny [4], and now, white supremacy [5,6]. Subsequently, the negative judgment and devaluation of others whose communities were intact prior to intrusion have suffered from the forces of assimilation and genocide. In the effort to dominate, Euro-Americans who adopted and projected a sense of superiority failed to understand the cultural context of the wide range and diverse cultures already in the Americas. In doing so, Euro-Americans lost the opportunity to selectively acculturate to cultures already in place. Subsequently, those who refused to become part of a functional and collective pre-existing whole have become the frayed threads that impair efforts to become a harmonious community. The frayed thread, white supremacy, if left unattended, will be the unraveling of a nation. To this end, we provide an epistemological orientation of the sacred circle of humanity and draw a connection between the impact of cultural colonization that has compromised the sacred circle of relationship to the adoption practices and policies impacting BIPOC and specifically Native American children. Specific examples related to the unraveling provide context for how the strain has impacted Native American culture and which set precedence for the ways in which humanity, the whole, within the United States continues to suffer from the strain of a loose thread.

## 2. Community, Colonization, Culture, and Divisions

“Life, Love, and the Pursuit of Harmony” [7] (p. 462) provides a holistic Native American cultural context that encompasses living in relation to all things. This orientation toward life accepts rather than rejects and includes rather than excludes. The nature of a people who seek to value all living things and weave all elements, regardless of how incongruent to the mean, provides a template for ways of being in a world filled with disharmony. In many Native American traditions, the Circle is a powerful symbol. Garrett & Portman provide the significance of the Circle as “a symbol of power, relation, peace, and unity” [7] (p. 457). In this tradition, people stand at the center of the circle, symbolic of the relationship between humanity and the world’s living creatures as well as their role in preserving tranquility and stability [7] (p. 457).

In essence, Locust recognizes that in the pursuit of harmony it is understood that well-being means not harboring “disharmony that is caused by suppressed anger, frustration, heartache, or fear” [8] (p. 13). Widely recognized today is that when we have trauma and other experiences that have not been processed and resolved, they can manifest into physical and mental health symptoms. One’s capacity to engage with others with an open heart and to be “in relation” is compromised when fear and judgment clouds one’s view of the world [9]. This clouded perception also compromises the way in which we engage in and with society. The fear-based projections from white supremacy continue to inform the existential reality of our society, particularly when people who inform policy have yet to resolve their own trauma [5,6].

Community is defined by a sense of belonging to a particular group of different people who share similar perspectives, experiences, or locations [10]. Membership in a community is drawn along physical and conceptual boundaries that develop individual identity. Like the threads that combine to form a piece of fabric, community membership is closely tied to cultural background within the definition of the characteristics that bind individuals to each other within that group. Sometimes these traits fall along ethnic, gender, race, language, or professional identities. Most often, the diverse personal traits of each individual have a stronger capacity to define their ascribed role in a community, strengthening the whole like interwoven strings of thread in the larger fabric. The individual attributes they develop later in life outside the community due to education or professional training become appliques or beads on a piece of fabric. 

For those who possess an indigenous identity that does not conform to the culture that is attempting to dominate, these embellishments often require abandoning parts of their native identity. Global colonization has historically induced assimilative processes upon native people as an arm of domination. These social tensions fray the individual threads of indigenous community fabric until it rips. Thus, belonging to a non-mainstream community has the almost certain outcome of limited opportunities to thrive because of the institutionalized constraints that exclude historically marginalized community identities. The close bond between individual identity, culture, and community calls for mental health practices to be redefined to move away from Eurocentric, colonizing practices. Such practices continue to ignore the personal identities of individuals and their bonds to the larger fabric, thus contributing to the cyclical unraveling of communities. 

To this end, mental health practices should be inclusive of the individual and their community/identities. This, as stipulated in the Social Justice and Multicultural Competencies, in which mental health professionals are expected to not only recognize their own biases but to do no harm to their clients [11]. This means having foundational knowledge of the culture and ways in which people embody their own sense of well-being. As discussed, being spiritually balanced and in harmony is central to BIPOC, and when disrupted it can unravel an entire community. What follows are several historical examples of how this begins to fray our collective society, and manifest as perceptions of mental health issues across generations. 

## 3. The Unraveling

In 1888, Mary Dissette, a Presbyterian mission school teacher assigned to the Native American community of Zuni, Arizona made note of her interaction with a respected member of the community by the name of Kwiwishdi. Culturally, Zuni’s accepted non-binary gender identity and referred to “alternative” gender as lhamana. In Zuni culture, Ihamanas are men who dress as women and who perform women’s work. They play important roles in culture and ceremonies [12]. Roscoe notes that Zunis “viewed gender as an acquired rather than an inborn trait [12]. Biological sex did not dictate the roles individuals assumed. Nor did Zuni thought limit gender to only two versions” [12] (p. 22). Given this cultural system that incorporated and valued alternative gender identity, fluidity and variance in dress and roles were accepted.

Dissette lacked insight into Zuni community fabric so sought to understand Kwiwishdi’s behaviors that were not consistent within her own worldview. Her adopted child, Daisy, was cousin to Kwiwishdi so she asked Daisy to interpret for her. Kwiwishdi explained that he wore women’s clothing in alignment to the type of work he performed, work that was associated with the female gender [12]. However, Dissette did not understand this perspective because she did work associated with the male gender and did not wear men’s clothing. Daisy then translated “he says you do not love all peoples in the world as much as he do[es], and that’s why he do[es] that” [12] (p. 24). After this exchange, Dissette judged Kwiwishdi’s behavior and perspective as an abstract concept common among his people [12]. 

The contrast in worldviews is revealed in this exchange. Most certainly, Dissette was culturally bound to a worldview in which people wear clothing according to the standards established in Europe. This view, being imposed upon the fabric by Dissette as someone who was frayed from the community, undermined Dissette’s capacity to accept Kwiwishdi for the way in which Kwiwishdi chose to dress, though the manner of dress had no bearing on Dissette. Worse yet, Dissette imposed a negative value upon Kwiwishdi’s choice of clothing as it did not conform to Dissette’s culturally bound, as well as limited worldview.

Certainly, labeling Zuni people as “spiritually arrogant” and referring to them as “creatures” [12] (p. 24). reveals, more so, Dissette’s arrogance and negative judgment. In essence, Dissette frayed herself from the whole as she, in her judgements projected onto Kwiwishdi, demonstrated what Menakem refers to as one of the past and present rules of white body supremacy in which “the white body deems itself the supreme standard against which the humanity of all other bodies is measured and judged, both structurally and philosophically” [5] (p. xi). In this way, Dissette untethered herself from a functioning community that embraced diverse ways of being and valued non-binary identities. Kwiwishdi recognized and called out not only the lack of love in Dissette’s worldview but, in this exchange, we begin to see the inception of disharmony imposed upon the Zuni way of life. This type of disharmony is created by the inability to accept and acknowledge pluralistic manifestations of identity that do not conform to a dominant norm or perception. 

Yet, when it is recognized that people are diverse in their attributes and capabilities, and they are included in the fabric of the community, then they, too, can feel valued. Their contributions, whatever they may be, give them a place of value and meaning. Locust recounts a story that demonstrates, once again, the attempt to unravel a culture that had already meaningfully incorporated all members into the circle and thereby enhanced a sense of well-being for all [8]. In the story, a man from the Hopi Tribe shares the story of a mentally challenged friend by the name of “Bear” who was described as “big and loving.” Bear’s essential duty to the village was to serve as the water carrier; however, “the Bureau of Indian Affairs social worker insisted that Bear go to a school in the city” [8] (p. 13). While attending the school, he reacted aggressively because he missed home. As a result of his reaction, Bear was committed to a state mental hospital, and after two decades returned home only to die [8]. This story illustrates the effects of removal from a familiar environment where an individual feels safety, belonging, and utility.

Bear, prior to the disruption caused by removal from his indigenous environment, was a meaningful and valued member of his community who depended on his contributions to the whole. Yet, the outside interference created disharmony with the community, an unraveling, and, ultimately, disharmony within Bear. The degree to which diverse ways of being have been rejected and violently imposed upon by elements of humanity who resist their own integration into the whole, will ultimately lead to the fall of all. This will be further considered as we move into greater discussion of the perils of our threads pulling away at the fabric.

The social tendency to outline conceptual lines or borders between members of the dominant culture and “others” within a particular society, perpetuates the systemic othering of historically minoritized communities and cultures (e.g., race and poverty) [13,14]. Culture is often erroneously used interchangeably with race. Regardless of term, difference in culture or race from the social norm leads to misinformation and misinterpretation of the needs of different people. Zaretta Hammond clarifies that culture is how we make sense of the world as humans regardless of race or ethnicity [15]. She identifies three levels of culture; “surface, shallow, and deep” [15] (p. 22–23). Prior to colonization, which led to racial division, indigenous communities were collaborative and fluid. Unlike African American, Latinx, Native American, and Pacific Islander communities, Euro-American culture centers on individualism [15]. This focus away from the whole causes disharmony in BIPOC communities. 

As a result of colonization, communities were transformed from fluid harmonious spaces that allowed for ambiguous identity and collectivism to spaces that were confined along cultural, racial, ethnic, gender, and linguistic boundaries. Male Eurocentrism and gender binaries were established as the norm across much of the colonized land and anyone who did not fit the dominant culture was “othered”. Sims-Schouten and Gilbert state that “’othering’ is achieved through three distinct representational pathways: through representational absence, through representations of difference, and through representations of threat” [14] (p. 86). Furthermore, “self-other distinctions are central to social and temporal spaces and identities, and research shows that specific social groups (such as members of minority ethnic communities) are often presented as the ‘other’” [14] (p. 86). In the stories of Zuni and Bear, “othering” caused the traditional cultures, communities, and its members to begin to unravel. 

The effects of colonization have been far-reaching, impacting the way in which most institutions were established and continue to exist in present day structures. Education, health, justice, and government institutions are the most common examples of social structures that continue to uphold elements of colonization that impact the way non-male, non-White, non-heterosexual, non-Christian people experience the world. Gloria Anzaldúa writes about how feelings of self-identity for women of color against a normed identity surface as a lack of safety and fear [16]. This trauma transcends generations and continues to define feelings of safety and belonging in BIPOC communities. In her description of the three levels of culture, Hammond asserts that deep culture “is what grounds the individual and nourishes his mental health. It is the bedrock of self-concept, group identity, approaches to problem solving, and decision making” [15] (p. 24). Being “othered” [13,14] then would be cause for disharmony that leads to social classifications of mental health imbalance. The complexities of belonging to various minoritized groups (e.g., women of color, non-binary, etc.) amplify the stressors and pressures that may lead to labeling of mental health imbalances. These complexities are not the fault of the person who is labeled by society and confined to specific groups. Instead, the responsibility rests with the social systems, structures, and practices that label people as different from a socially established norm.

## 4. The Next Seven Generations; Our Children

*“Children learn from what they see. We need to set an example of truth and action.”*

—Howard Rainer, Taos Pueblo-Creek [1]

Systems and policies that are meant to protect our children and families are instead harming them through forced acculturation and by enforcing white Eurocentric perceptions of how families should be. Families of color are often held up against Eurocentric standards without consideration of differences in culture and ways of knowing. Moreover, people who have experienced centuries of systemic racism and othering are looked down upon when their lives show evidence of such racism. Where this harms our children the most is in so-called child welfare services where actors are charged with protecting children’s best interests. In reality, best interests are often judged from an individualistic Eurocentric lens and without taking into account the effects that centuries of racism has had on people of color. This is evident in many facets of child welfare beliefs and practices, including definitions of good parenting, ignorance about attachment theory, misunderstanding of cultural practices, and punishment for poverty.

When Black, Indigenous, and parents of color are evaluated by the accepted Eurocentric standards and white norms, they risk having their children taken from them [17,18]. Native children have been removed from their families simply for practicing their cultures, and parents of color have been deemed unfit and not good enough to care for their own children [17,19]. In the United States, Children of color are disproportionately represented in foster care, and racial disparities exist along the continuum of the child protection process. Children of color are overrepresented in maltreatment reports, investigations, and removal from their homes [20,21,22]. Black children are overrepresented in reports of child maltreatment [21], and a higher percentage of Black families experience investigation for maltreatment [20]. Native American and Black children are at the highest risk of being placed in foster care [22]. Parents of Native American children are at the highest risk of losing their parental rights [23]. These disparities are due to prejudice and ignorance of cultural practices that are different from Eurocentric standards.

Overrepresentation of Native American children in the U.S. child welfare system has roots in the boarding schools and the Indian adoption project (both of which aimed to rescue children from their own cultures) [24]. The attempt to assimilate and acculturate Native American children and save children of color from their families continues today. Those who would save children perceive cultural differences as barbaric and uncivilized practices that need to be stopped. Thus, there are still some transracial and even international adoptions in which white families attempt to “rescue” children from their homes [25,26]. Contrary to these beliefs, children of color do not need to be saved from their families and raised according to white standards [19].

The effects of white supremacy are not limited to the United States. In Canada, Indigenous foster children have been kept from their biological parents due to misapplication of attachment theory. That is, parents have completed successful rehabilitation and maintained contact with their children throughout their time in care, only to have Canadian courts deny their applications to have their children returned to them [27]. Decisions are based on the precedent of a child protection case from 1983 [28]. In this case, attachment theory was considered to be more important than culture and cultural preservation when deciding on the best interests of an Indigenous child. Based on this case, many courts have ruled against Indigenous children’s biological parents, stating that, according to attachment theory, returning children to their parents would harm them (see, e.g., [29]). However, courts in Canada are misapplying attachment theory when it comes to Indigenous peoples. Eurocentric theory of attachment to one or two primary caregivers does not apply equally to all families. For example, in Blackfoot culture, parents are just one part of a cultural network. Children are nurtured and parented by the community more so than by individual parents [30].

Two sides of poverty, both the assumption that poverty exists when it does not and the true existence of poverty imposed by racism, are more examples of a frayed fabric caused by White supremacy. One the one hand, children are removed from their homes and placed with white families because those in the child welfare system do not respect or understand cultures in which the collection of possessions does not define success. Many Indigenous peoples practice a culture of reciprocity, living within their means, giving and sharing to benefit the whole rather than the one (e.g., [31,32]). When these cultural practices are misunderstood, communities are labeled as “poverty stricken” when they are not. The collection of material wealth is not consistent with many Indigenous nations’ way of life. Thus, Native peoples who are living in a way that fits within their value system are judged and found wanting by those who do not understand these values [19].

On the other side, children are removed from their homes and placed with white families because of their poverty, which was the result of colonization, attempted erasure, and systemic racism. Poverty does exist among people of color. Slavery, Jim Crow laws, red-lining, eugenics, and forced removal from tribal lands are but a few of the ways that white supremacy has forced people into poverty and sought to keep them there [19,33]. Is it right to subject a people to poverty and then take away their children because of such poverty?

Children who are placed into foster care face additional trauma if their foster families are culturally ignorant. Children may feel like they do not fit in because there is no one in their community who is like them [34]. Black girls experience loss of identity and self-esteem when foster parents do not understand the cultural significance of Black hair [35]. Once children are adults, they can lose a sense of belonging because they know little about their ethnicity and culture [36,37]. In the United States, the Indian Child Welfare Act (ICWA) was created in response to the large number of Native American children who were being removed from home and placed in families far from their Native culture. This is something that should be considered for all minoritized children in foster care.

Placing children of color with white families without first attempting to place them with their own people is a continuation of colonization and systemic racism and rests on the assumption that White understanding of families and parenting is correct. This practice systematically dismisses people of color and their cultures and compounds historical trauma. In their review of the Racine v. Woods [28] case, Choate et al. [27] argue that placing Indigenous children in white foster homes far from their Indigenous culture is worse than what happened in the infamous boarding schools. Although boarding schools were abhorrent, children were with other indigenous children, whereas children placed in white foster homes are typically removed from their culture entirely. Once again what we see is that the removal of Native American children from their families and placing them into care far from their cultures can be likened to a new Trail of Tears. This being the continuance of policy that hastens the unraveling of human interconnectedness. 

## 5. The Cost of Othering

When disparities that “other” groups of people are called out, they are often met with comments about resilience and grit [14]. These are common concepts ascribed to historically minoritized groups as necessary traits that they lack when viewed through a deficit-based lens. Eurocentric, colonizing perspectives utilize a deficit-based lens to call out perceived deficiencies in minoritized communities as an integral practice of othering. However, the reality is that there are numerous examples of resilience and grit within minoritized communities and cultures. Non-traditional examples of resilience can take the form of “resisting bad treatment and racism, as well as reflecting agency, identity and ownership of one’s own life and choices” [14] (p. 87). Through the lens of dominant social structures, these alternate examples of resilience are perceived as aggression, anger, and general dysregulation.

Alternate expressions of resilience are often weaponized against historically marginalized groups to establish difference and threat requiring rehabilitation or treatment [14]. In the same article about redefining resilience, Sims-Schouten and Gilbert assert that structural racism is not considered in the practice of mental health and other health professions [14]. In fact, they explain that racism and biased definitions of resilience and “othering” are what lead to the idea that members of minoritized communities need to develop resilience. The oversimplified notion that individuals need to rise in the face of adversity in socially prescribed ways minimizes and invalidates the complexity of the varied traumas experienced by historically minoritized groups, many of which are generational traumas.

Psychotherapist Resmaa Menakem writes about the effects of racialized trauma on the human body in his book *My Grandmother’s Hands* explaining that all forms of trauma interact in ways that cause increased harm, as new traumas are experienced and trigger past traumas [6]. He asserts that intergenerational traumas are those which transcend and extend from one generation to the next and are rooted in historical aspects of an individual’s identity and experiences as members of a particular group of people (e.g., racial, ethnic, gender, linguistic, etc.). These are traumas imposed by systems and institutions that were never meant to include them [6]. The human body experiences the world as a whole. The body systems react to stimuli in ways that are absorbed and processed collectively as emotions, reactions, and stress [6]. While certain memories or experiences may trigger specific reactions or feelings, the body collects these various reactions and accumulates them in a way that creates sediment. These reactions and experiences are compounded when the nature of the trauma originates from identity because of the organic origins of identity and an individual’s connection to identity, this type of trauma is magnified when the entire fabric of society is structured to re-traumatize.

## 6. Do No Harm

Cultural competence, in the field of education, is defined by Lindsey et al. as the ability of educators to engage interactively within diverse settings in ways that acknowledge, respect, and affirm cultures that are different from their own [38]. Due to the nature of the work a mental health practitioner is engaged with, they should go to great lengths to ensure that they are not causing further trauma on their clients by using subtractive measures that ignore, are blind to, invalidate, or devalue the cultural identities of each individual. An example of this was described in the interaction between Dissette and Kwiwishdi [12], and also in Bear’s experience [8]. These two examples speak to the imposition of a culturally encapsulated worldview attempting to unravel a functioning community as well as an individual’s sense of safety and belonging within that community. For this reason, developing cultural competence requires a willingness to listen actively to the people with whom one interacts in ways that allow mutual learning to gain a better understanding of the lived experiences of the other.

Active listening is a skill that additionally requires a practitioner to acknowledge they have personal biases based on their own experiences that may influence the way they interact with others. In the case of educational leaders, Tollefson and Magdaleno identify an acknowledgment gap that can influence a leader’s ability to provide or deny opportunities to students based on a leader’s failure to acknowledge their own biases and how those limit or enrich the students they serve [39]. In mental health, the failure of a mental health practitioner to acknowledge how their own perceptions and experiences about members of other communities outside the cultures they identify with can also inhibit the services they provide their clients. Researchers suggest that cultural competence is an integral part of a mental health practitioner’s training [40]. Yet, the application of cultural competence in mental healthcare within indigenous communities requires further discussion. 

## 7. Cultural Wealth and Mental Health

Tara Yosso speaks of community cultural wealth as the cultural knowledge that family, social, aspirational, language, resistance, and navigational resources provide [41]. She discusses how the education system has relegated non-white, non-English speaking students to a second-class citizen status. The institutionalized practice of centering whiteness and English as the dominant cultural norms in education continue to cause trauma to students who do not identify with those social markers. Professional practices are racialized and politicized across industries including physical and mental healthcare contributing to the continued unraveling of communities. 

Cultural change is often suggested as necessary for success in socio-political contexts. Some researchers suggest that individuals who leave their indigenous communities are more likely to voluntarily give up their cultural traditions to embrace practices that will place them at a greater advantage to thrive in their new contexts [42]. The Reyes-Garcia et al. study centered on indigenous Amazonian cultures and their knowledge of the use of plants within their society [42]. They looked at cultural changes over time and factors that contribute to change in traditional knowledge. Their article contends that this type of knowledge is in decline among global indigenous cultures. Cultural changes in traditional knowledge were less apparent in remote communities than in communities that were in proximity of towns engaged in commercial and political interactions [42]. Through their study, Reyes-García et al. explain the impact contemporary mainstream social structures can have on indigenous cultures over time [42]. 

A similar study on traditional ecological knowledge in rural Spain found that traditional knowledge about agriculture declined due to the integration of modern farming practices however, researchers also determined that local identity in rural protected ecological areas is defined by traditional methods of livestock farming [43]. Such cultural change points to how transcultural interactions in the interest of survival [42] and economic gain [43] can impact loss of traditional practices and knowledge. However, Reyes-Garcia et al. point to choice rather than encroachment as the driver for change in indigenous communities thus placing the responsibility for loss of traditional practices on indigenous people [42]. Resiliency then is ascribed to BIPOC as a means to adapt to challenges and changes in ways that ensure their survival [14]. 

Furthermore, Cox contests the role of traditional knowledge in modern contexts as one of mutual benefit whereas scientists gain from the traditional knowledge of medicinal plants while indigenous cultures gain from the protections that come with conservation of biodiversity [44]. This perspective encapsulates the idea that indigenous natural resources are to be protected for the benefit of advancing scientific discovery. While Gómez-Baggethun et al. acknowledge the link between traditional knowledge and local identity [43], neither study accounts for the impact the loss of cultural knowledge has on perceptions of indigenous self-identity and connection, and how these come to affect indigenous mental health.

According to Desai et al., the organizational culture of mental health entities may be a barrier to mental health access for patients whose cultures are different than that of its practitioners [40]. The structure of mental health organizations prevents practitioners from fully engaging with historically minoritized communities. Through their study of community health practices in Puerto Rican communities in New York and Puerto Rico, Zerrate et al., found that these barriers could be diminished or eliminated by establishing connections with community healers such as espiritistas and santeros [45]. The process of relationship building with community healers may be the key to developing experiential knowledge for mental health practitioners that is far more valuable than cultural competence alone.

The Zerrate et al. findings point to the importance of community-based collaborations to provide access to community mental health services through well established and trusted individuals within communities [45]. Additionally, the study highlights the knowledge that traditional mental health services merged with culturally based health services can work to support historically minoritized communities. Through the incorporation of traditional knowledge [42,43], and cultural wealth [41], mental health practitioners build on their existing practices to incorporate relevance and representation of BIPOC communities. 

## 8. Conclusions

Acculturation and othering along with forced assimilation and alignment with Eurocentric values has fomented historical and generational trauma in communities who have experienced targeted attacks [32]. However, mental health practitioners can combat white supremacist practices and foster well-being in communities so that humanity with its wide range of diversity (i.e., neurological, gender identity, etc.) can thrive. Recognizing the debilitating impact of cultural invasion [46] the conscious restoration and respect for the many ways humanity exists ultimately strengthens the fabric of this nation. In doing so, a humanist approach to mental health is achieved. 

Enculturation, a process by which individuals learn about their own ethnic minority culture and engage in cultural practices in their everyday lives [47], provides the opportunity to restore what has been subsumed or lost during colonization. Enculturation and cultural assets can buffer against trauma and improve mental health [48,49]. Moreover, when people have a stronger ethnic identity, they are happier, better able to cope with stress, more optimistic about the future, have higher self-esteem, and have better mental health [32,47,48,49,50]. Incorporation of cultural practices, exploration of self- identity to native communities and across various contexts can strengthen mental health practices in support of improved care for BIPOC. To the extent that we can elevate the mental fortitude of our children to understand the value of all people, we strengthen the fabric of this nation.

The European diaspora has sent reverberations across this land generated by people fleeing a continent gripped by war, poverty, epidemics, misogyny, religious persecution, and authoritarianism [51]. In understanding how trauma has been projected out onto this land and the original nations, those who have descended from those seeking refuge can gain a greater appreciation for their ancestors seeking a better life while also resolving the vertical manifestations of embodied trauma [6]. Healing their own generational trauma would allow those descending from the European diaspora to become an equal and stabilizing thread of the community fabric. In turn, reflective mental health practices that seek to re-establish societal interconnectedness require the application of knowledge gained from authentic engagement with BIPOC communities. Through our discussion, we have illustrated the need for greater focus on the sense of individual and community identity, connection to society, and authentic engagement with BIPOC, as well as other historically marginalized communities to inform community mental health research and practice. This could become a reality once those who inherited the trauma of their ancestors during the European diaspora have resolved their own embodied trauma. 

In conclusion, the people fleeing a continent gripped by war, poverty, epidemics, misogyny, religious persecution, and authoritarianism [51] carried embodied trauma which was then projected out across this land [6]. Understanding the impact of the European diaspora reveals a chronic state of dysregulation that is grounded in a fear-based worldview and that is easily triggered by perceived threats. Once this is fully recognized and attended to then the U.S can address its weakest strand. In healing the vertical manifestations of generational trauma, the individual threads of the nations’ fabric will not only exist equally but through its interdependence, strengthen the entire fabric, and ultimately create harmony for future generations.
